# Assessing the invasive risk of Rhinotermitidae in China under current and future global warming scenarios using the MaxEnt model

**DOI:** 10.1186/s12983-026-00600-x

**Published:** 2026-02-21

**Authors:** Riaz Hussain, Lianxi Xing, Yuan Hua

**Affiliations:** 1 College of Life Sciences, Northwest University, Xi’an, 710069 China; 2 Shaanxi Key Laboratory for Animal Conservation, Northwest University, Xi’an, 710069 China; 3 Key Laboratory of Resource Biology and Biotechnology in Western China, Ministry of Education, Northwest University, Xi’an, 710069 China

**Keywords:** *Coptotermes*, *Reticulitermes*, Climate change, Distribution pattern, MaxEnt model, Control

## Abstract

**Background:**

Biodiversity and distribution patterns are essential components for ecological and biogeographical research. The family Rhinotermitidae (sensu lato; *Coptotermes* and *Reticulitermes*) is among the most detrimental and widespread termites in China, causing severe damage to the ecosystem. However, their geographical distribution patterns and species richness hotspots are little comprehended, posing substantial challenges for successful management and control initiatives. After cleaning, along with bioclimatic variables, we uploaded 215 occurrence records for *Coptotermes* and 184 for *Reticulitermes* to the MaxEnt model to forecast their risk habitats during the Current (1970**–**2000) period and under prospective global warming scenarios.

**Results:**

We found that *Coptotermes* are mainly distributed in southern China, while *Reticulitermes* are primarily found in southern China and the Qinling Mountains. The hotspots of *Coptotermes* are primarily located in Guangdong Province, while those of *Reticulitermes* are located in Hubei and Guangdong Provinces. Annual mean temperature (Bio1; 73.2%) is mainly responsible for the distribution of *Coptotermes* in China, while mean diurnal range (Bio2; 31%) and precipitation of driest quarter (Bio17; 31.4%) are mainly affecting the distribution of *Reticulitermes*. The MaxEnt model exhibited outstanding performance for *Coptotermes* (AUC 0.955; TSS 0.808) and *Reticulitermes* (AUC 0.944; TSS 0.732). Under climate scenarios from 1970 to 2000, the total risk areas of *Coptotermes* and *Reticulitermes* were 0.73 million km^2^ and 2.25 million km^2^, respectively. Under SSP2-4.5 scenarios, areas classified as negligible-, moderate-, and high-risk are expected to expand and shift towards northern China in the future, leading to a rise in Rhinotermitidae (sensu lato) population size. Therefore, it indicates a serious threat to infrastructure, crops, and agricultural systems.

**Conclusions:**

This research enhances our knowledge about the present geographic distribution and species richness hotspots of *Coptotermes* and *Reticulitermes* in China and the potential impact of future global warming on their distribution and shift towards novel habitats in southern and northern China. Therefore, this study aids in the implementation of control and early prevention strategies in high-risk regions.

**Supplementary Information:**

The online version contains supplementary material available at 10.1186/s12983-026-00600-x.

## Background

**Fig. 1:**
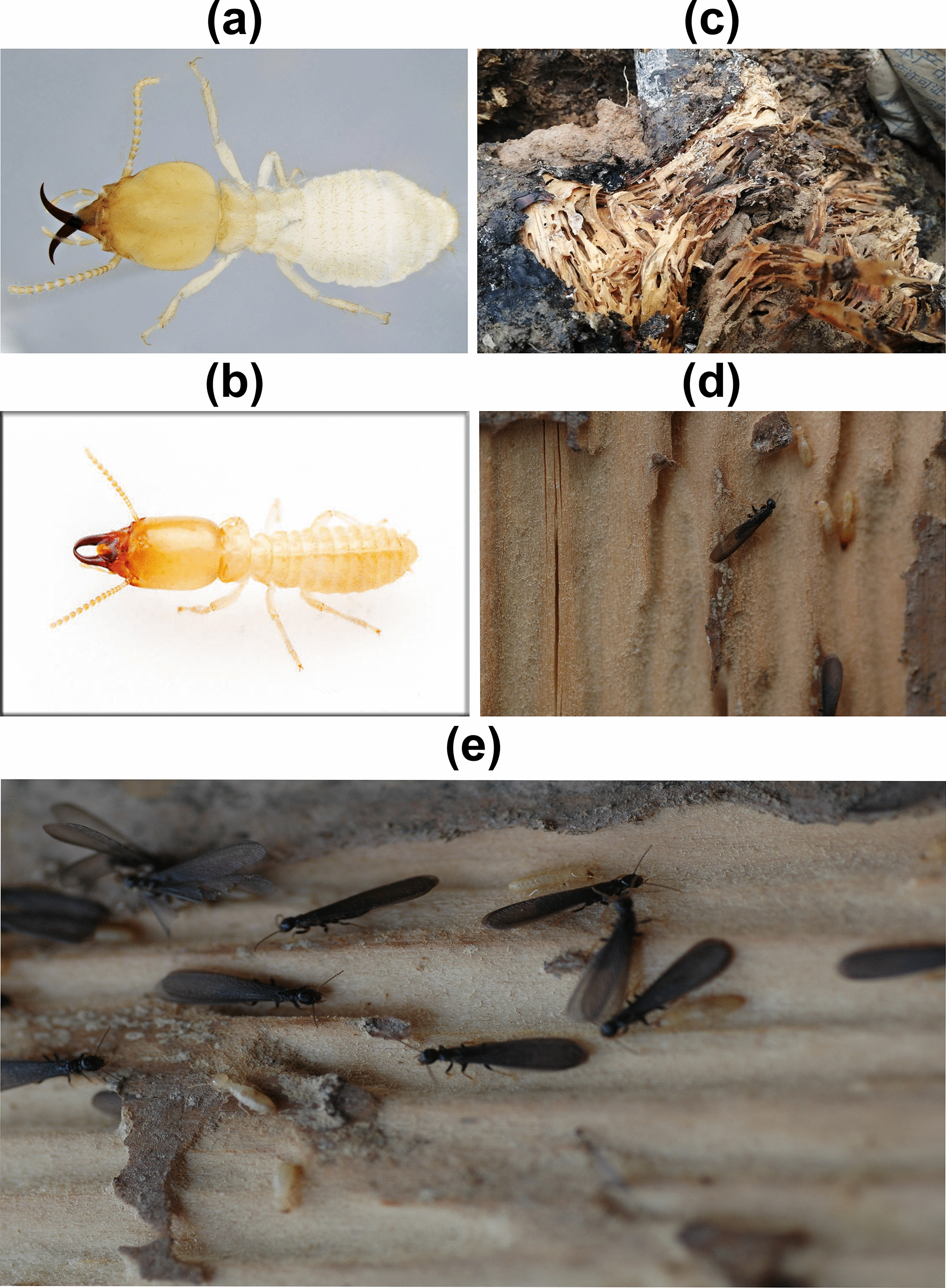
Rhinotermitidae termites and associated damage in China: **a** representative species of *Coptotermes*; **b** representative species of *Reticulitermes*; and **c**–**e** example of wood damage caused by Rhinotermitidae termites. Pictures of wood damage were taken in the field

Termites are social insects belonging to the order Blattodea [1, 2]. The family Rhinotermitidae (sensu lato) [3, 4] has 15 genera and over 300 described species globally, known as subterranean termites because of their subterranean lifestyle [1, 5]. Rhinotermitidae are a late-diverging lineage within the non-Termitidae termites and are phylogenetically positioned close to the origin of Termitidae [1, 6]. Up-to-date phylogenetic studies show that Rhinotermitidae, as previously defined, is not monophyletic, with main genera such as *Coptotermes* and *Reticulitermes* now placed within Heterotermitidae [3, 4]. However, because the term "Rhinotermitidae" has been widely used in research, we employed Rhinotermitidae (sensu lato) throughout this study to ensure taxonomic clarity. The total number of reported Rhinotermitidae (sensu lato) species and genera throughout China is 185 and 7, respectively [3, 4, 7]. Termites are particularly distributed in southern China, causing heavy damage to the ecosystem. *Reticulitermes* and *Coptotermes* species are said to cause a broad spectrum of harm to the trees, agriculture, and human infrastructure (Fig. 1) [8, 9]. According to national-level assessments, affecting more than 40% of the total land area in China, termites have caused damage to between 30 and 90% of structures in the regions south of the Yangtze River, resulting in direct economic losses of 2 to 2.5 billion RMB per year [10, 11]. The structural reliability, historical conservation, agricultural, and forestry sectors in China face significant issues due to these termite taxa's cryptic hunting behavior, soil contact, wood-eating preferences, and the capacity of vast colonies to remain unnoticed [9, 12–16].

Termites live in nests that provide food, shelter, and optimal conditions for their survival and protect them from severe temperature and drought, which aligns with their behavior and physiology [2]. Termites do not actively migrate like other animals; instead, they expand their territory through local colony growth with the help of making secondary nests and satellite colonies and by dispersing over long distances (*Coptotermes formosanus*: ~1300 m; *Reticulitermes flavipes*: ~458 m) through seasonal swarming of winged reproductive alates, which is the main way of new colony establishment following nuptial flights [17–20]. They change their foraging behaviors in response to climate-related factors, particularly temperature and humidity variations, which made them ideal for climate change studies [12, 21]. Research on Rhinotermitidae (sensu lato) has primarily focused on their taxonomy, evolutionary biology, and behavior, while less attention has been given to wide-scale ecological research on finer taxonomic levels [1–4, 17].

Temperature and humidity significantly influence the geographic distribution of termite species [12, 17, 22–25]. Variations in moisture and temperature sensitivities between the stages of growth allow some termite species to adapt to several specific environments, enhancing their climate endurance and invasion capability [24]. A previous study examined the influence of temperature and humidity on long-term survival of termites, such as *C. formosanus* and *R. flavipes*. Both termite species preferred low to moderate temperatures and elevated humidity, with death rates escalating significantly under hot or drier conditions [21]. The worldwide distribution analysis of *Coptotermes gestroi* indicates that this species flourishes in warm, moist, and highly urbanized lowland areas, with its proliferation expected to intensify due to continued development and population expansion in tropical and subtropical regions [26]. Studies demonstrated that development, transportation, and human population were the drivers of the invasion and dispersion of numerous termite species in China [26, 27].

Due to persistent global warming, the range of termites is anticipated to extend into areas that were previously deemed unsuitable or marginal [17]. Elevated temperatures and modified precipitation trends can foster climatic conditions conducive to termite survival, reproduction, and colony development in previously unsuitable regions [12, 17, 21, 28]. Termites, as ectothermic animals, exhibit heightened sensitivity to temperature and moisture conditions, with even minor climatic changes capable of substantially affecting their activity and distribution limits [12, 21]. As global temperatures rise, areas formerly unsuitable may become conducive to termite colonization, enabling their expansion into higher latitudes [17, 28]. Ecological niche models (ENMs), particularly the maximum entropy (MaxEnt) model, are widely used to identify species-environment associations by identifying and modeling influential environmental factors that assess their appropriate habitats [29, 30]. Researchers widely used the MaxEnt model in different ecological and biogeographic work, such as control and management studies, to create habitat suitability maps [31–33]. Buczkowski et al. [17] and Liu et al. [31] used the MaxEnt model to predict termite diversity trends in China and worldwide.

One of the greatest structural pest hazards in China is termites (principally *Coptotermes* and *Reticulitermes*) [9–11]. These termites are found across mainland China's humid, low-elevation to subtropical climates, and they frequently live in both natural and artificial settings [9, 10, 17, 34]. However, extensive research investigating the geographic trends and environmental variables influencing Rhinotermitidae (sensu lato) in China is still limited. During current research, the MaxEnt model was used to assess the present and future distribution of Rhinotermitidae (sensu lato; *Coptotermes* and *Reticulitermes*) under climate change conditions to identify critical ecological variables and forecast possible habitat shifts in the future. This study is essential for pinpointing areas with the highest invasion risk and for executing proactive management strategies in the event of future invasions in China.

## Materials and methods

### Data collection

The distribution data for Rhinotermitidae (sensu lato; *Coptotermes* and *Reticulitermes*) were collected from the following reliable resources: field collection records; Global Biodiversity Information Facility (GBIF, https://www.gbif.org/), utilizing the “rgbif” package [35] in R v4.4.3 [36]; iNaturalist (https://www.inaturalist.org/), utilizing the “rinat” package [37]; and published literature (in English and Chinese). In this study, we selected the genera *Coptotermes* and *Reticulitermes* due to their destructive power (high economic impact) and availability of data. Following Dong et al. [34], termite-infested logs were gathered in the field, placed in plastic containers covered with damp towels, and routinely sprayed with water. They were kept in the laboratory at room temperature. Data from the above-mentioned sources were downloaded on September 25, 2025. We used Google Earth (https://earth.google.com/) to locate the sites described in the literature without coordinates. We compiled a total of 584 distribution records (281 for *Coptotermes* and 303 for *Reticulitermes*). The total number of species collected was 24 (Table S1). The R package “spThin” was used to perform spatial autocorrelation analysis to decrease the impact of model overfitting resulting from the over-closed (smaller than 1 km) distribution records [38]. After preprocessing, this study included 215 occurrence records for *Coptotermes* and 184 for *Reticulitermes*.

A total of 19 bioclimatic variables were downloaded for the Current (1970–2000) period. Future climate data were downloaded from the Coupled Model Intercomparison Project Phase 6 (CMIP6), released in 2021 [33, 39]. The global climate model (GCM)—ACCESS-CM2—was chosen. We projected the risk habitats based on future environmental variables under the SSP2-4.5 scenarios for the years 2050s (2041–2060), 2070s (2061–2080), and 2090s (2081–2100). The above-mentioned environmental variables were downloaded from the WorldClim database [http://www.worldclim.org (accessed on October 1, 2025)], with a resolution of 2.5′ (\~4.3 km).

### Model evaluation

The MaxEnt model was used to identify the contribution rates of 19 bioclimatic factors for both *Coptotermes* and *Reticulitermes* (Table 1). To mitigate the problem of overfitting, all 19 bioclimatic factors underwent correlation and principal component analysis (PCA) with the “cor” function in R v4.4.3 (Figs. S1, S2) [36, 40]. In the case of both genera, six bioclimatic factors that exhibited substantial contributions (as indicated in Table 1) between pairs of strongly correlated parameters (|r| > 0.8) (Figs. S1, S2) [32, 41] were selected for further study, while the other parameters were omitted from the subsequent analysis.

**Table 1:** Percent contribution of 19 bioclimatic variables used in the initial MaxEnt model

Variable	Description	*Coptotermes* Percent contribution	*Reticulitermes* Percent contribution
Bio1	Annual mean temperature	**64.4**	**0.8**
Bio2	Mean diurnal range [mean of monthly (max temp-min temp)]	**8.6**	**25.1**
Bio3	Isothermality (Bio2/Bio7) (×100)	0.5	3.1
Bio4	Temperature seasonality (standard deviation × 100)	0.5	0.6
Bio5	Maximum temperature of warmest month	0.1	0.1
Bio6	Minimum temperature of coldest month	1.6	**21.1**
Bio7	Temperature annual range (Bio5-Bio6)	**3**	**7.1**
Bio8	Mean temperature of wettest quarter	0.7	2.1
Bio9	Mean temperature of driest quarter	**4.5**	0.1
Bio10	Mean temperature of warmest quarter	1.4	0.3
Bio11	Mean temperature of coldest quarter	1.5	0.1
Bio12	Annual precipitation	**1.7**	6.6
Bio13	Precipitation of wettest month	1	**1.2**
Bio14	Precipitation of driest month	2.9	0.3
Bio15	Precipitation seasonality (coefficient of variation)	0.8	4
Bio16	Precipitation of wettest quarter	1.1	0.1
Bio17	Precipitation of driest quarter	**3**	**26**
Bio18	Precipitation of warmest quarter	1.2	1.6
Bio19	Precipitation of coldest quarter	1.4	0.1

Six variables (the bold ones) were used in the final MaxEnt model for predicting *Coptotermes* and *Reticulitermes* risk habitats, based on the substantial contributions between pairs of strongly correlated parameters (|r| > 0.8)

“ENMeval” was utilized in R v4.4.3 individually for *Coptotermes* and *Reticulitermes* to mitigate model overfitting by adjusting the configurations of feature combinations [linear (L), quadratic (Q), product (P), threshold (T), and hinge (H)] and regularization multiplier (RM) value (1–3) in intervals of 1, alongside the feature kinds (L, LQ, H, LQH, LQHP, and LQHPT), while calculating the Akaike information criterion (AICc) metrics utilizing “block” [36, 41, 42]. The feature class “LQ” and RM value “1” for *Coptotermes* (Fig. S3), along with “H” and RM “1” for *Reticulitermes* (Fig. S4), correspond to the model exhibiting the smallest delta AICc as selected in the MaxEnt model. A total of 10,000 backdrop points were chosen. Of these, 25% of the points were designated for testing, while the remaining 75% were allotted for training. The threshold criterion was set to equate training sensitivity and specificity. The selected replication type was cross-validation. The quantity of replicates was fixed to 10. The output file type was specified as cloglog [32, 33, 41]. The rest of the options were left unaltered. The risk habitats for *Coptotermes* and *Reticulitermes* were classified into four types: 0–0.2 negligible risk, 0.2–0.4 less risk, 0.4–0.6 moderate risk, and 0.6–1.0 high risk [32, 41].

The performance of the MaxEnt model was assessed using the area under the curve (AUC) and the true skill statistic (TSS) [32, 41]. The MaxEnt model yielded the AUC value. The ROC curve shows the true-positive rate (sensitivity) on the y-axis and the false-positive rate (specificity) at different thresholds on the x-axis. The AUC score was calculated by determining the area enclosed by the curve and the abscissa [32, 41]. The TSS value is calculated as specificity plus sensitivity minus one. According to the AUC value, the model's efficacy was categorized as failing (0.5–0.6), poor (0.6–0.7), fair (0.7–0.8), satisfactory (0.8–0.9), or exceptional (0.9–1). Based on the TSS value, the model's performance was categorized as undesirable (<0.4), acceptable (0.4–0.55), poor (0.55–0.7), satisfactory (0.7–0.85), or excellent (0.85–1) [32, 41].

### Analysis of species richness

A grid of dimensions 1 × 1 (roughly 100 km × 100 km) was used in R v4.4.3 [36] to show species richness patterns [41]. We used the “letsR” package [43] to generate a grid-based species presence (1) or absence (0) matrix from the point occurrence data, where rows represent geographic locations (grid cells) and columns represent species. The resulting matrix was then imported for further analysis into the EstimateS 9.1 software [44] by using the default options. The output was exported in the text format. The “ggplot2” package [45] was used for constructing a cumulative curve. This curve was used to evaluate the precision of the collected species count [41].

## Results

### Geographic distribution

**Fig. 2:**
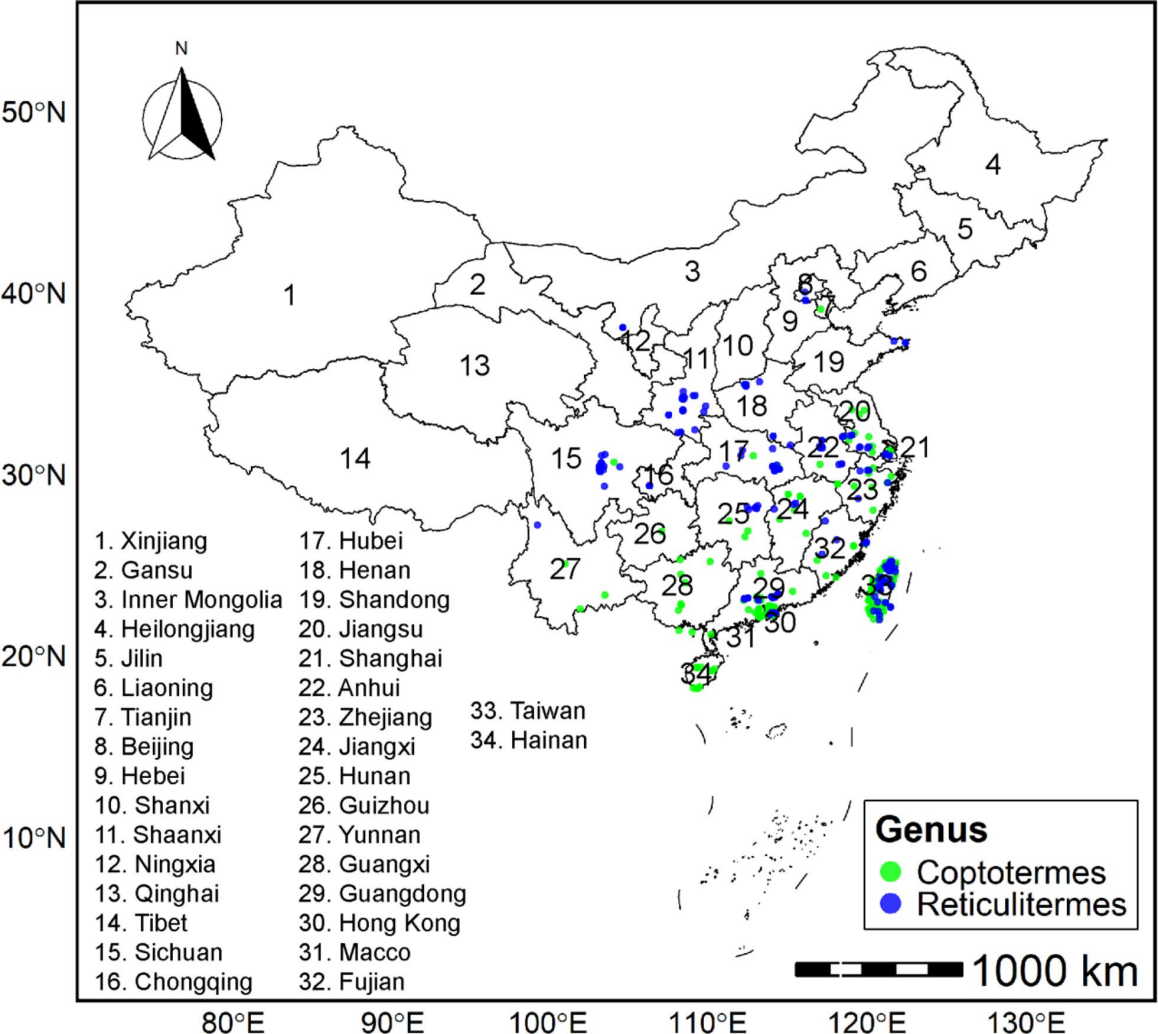
Geographical distribution of Rhinotermitidae (sensu lato) across China. The green color shows *Coptotermes*, while the blue color represents *Reticulitermes*

**Fig. 3:**
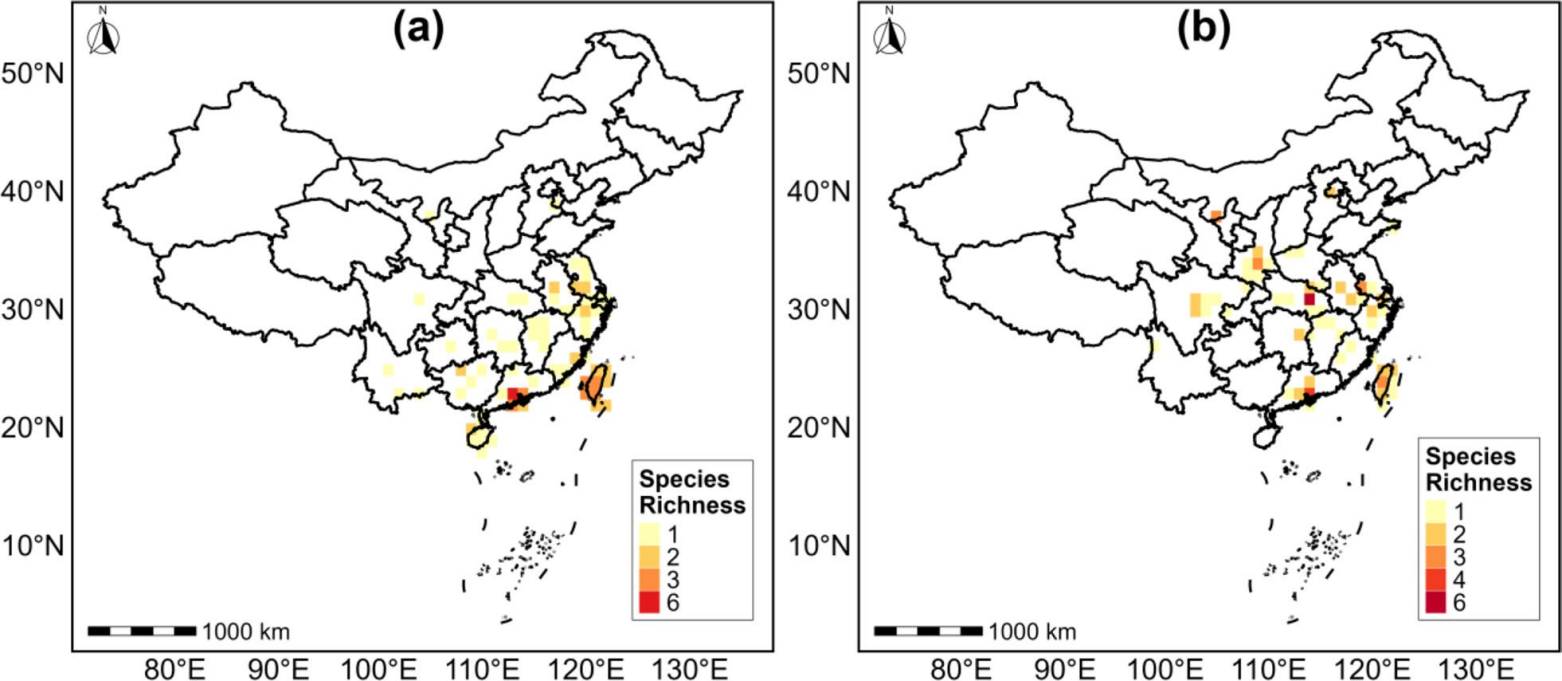
Rhinotermitidae (sensu lato) species richness patterns across China: **a** species richness of *Coptotermes* and **b** species richness of *Reticulitermes*. Dark red color represents grids with the highest species richness, while yellow color shows grids with the lowest species richness

The genus *Coptotermes* is predominantly distributed in southern China, while *Reticulitermes* is mainly distributed in southern China and the Qinling Mountains (Fig. 2). The species accumulation curve indicates that 24 Rhinotermitidae (sensu lato) species were compiled in the database; however, the bootstrap mean approach produced an estimate of 29 species. The percentage of gathered species was 83%, indicating that the majority of species were collected (Fig. S5). All species collected during this study are given in Table S1. Out of 24 species, 6 belong to the genus *Coptotermes* and 18 to *Reticulitermes*. The species richness of both genera exhibited an uneven distribution pattern. The highest species richness of *Coptotermes* is found in Guangdong Province (Fig. 3a), while that of *Reticulitermes* is found in Guangdong and Hubei Provinces (Fig. 3b).

### MaxEnt model validation and variable impact

**Fig. 4:**
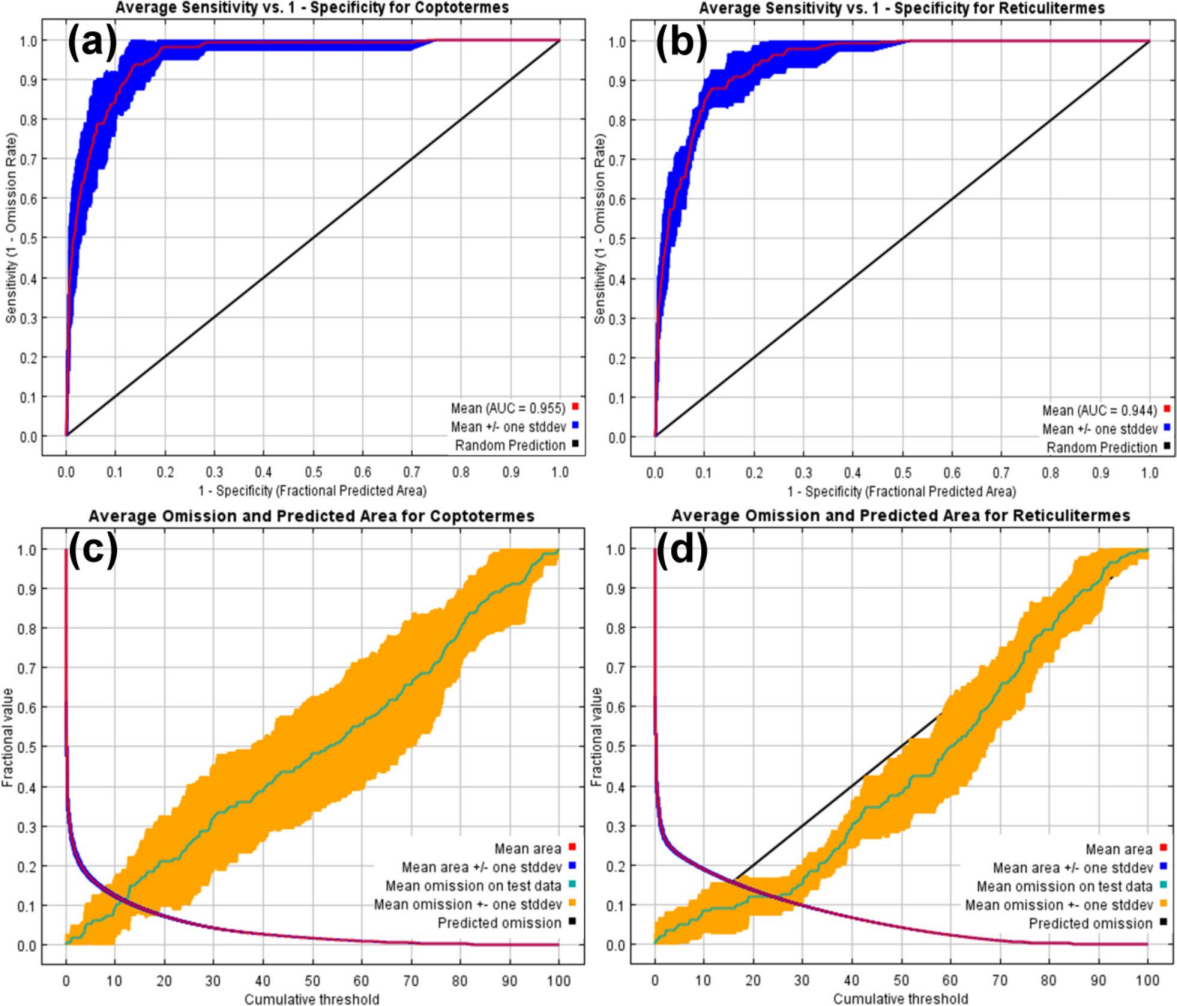
MaxEnt model outputs for Rhinotermitidae (sensu lato): **a** the receiver operator characteristic curve (ROC) showing the predictive accuracy of the model for *Coptotermes*; **b** the ROC illustrating the predictive accuracy of the model for *Reticulitermes*; **c** the omission and predicted area curve for *Coptotermes*; and **d** the omission and predicted area curve for *Reticulitermes*

The results indicate that the model used in this experiment displayed robust performance, evidenced by an AUC value of 0.955 for *Coptotermes* (Fig. 4a) and 0.944 for *Reticulitermes* (Fig. 4b). The TSS values calculated for *Coptotermes* and *Reticulitermes* were 0.808 and 0.732, respectively. The average omission and predicted area curves for both genera showed that the predicted omission rate closely aligns with the average omission, further highlighting the model's strong performance (Fig. 4c, d).

**Fig. 5:**
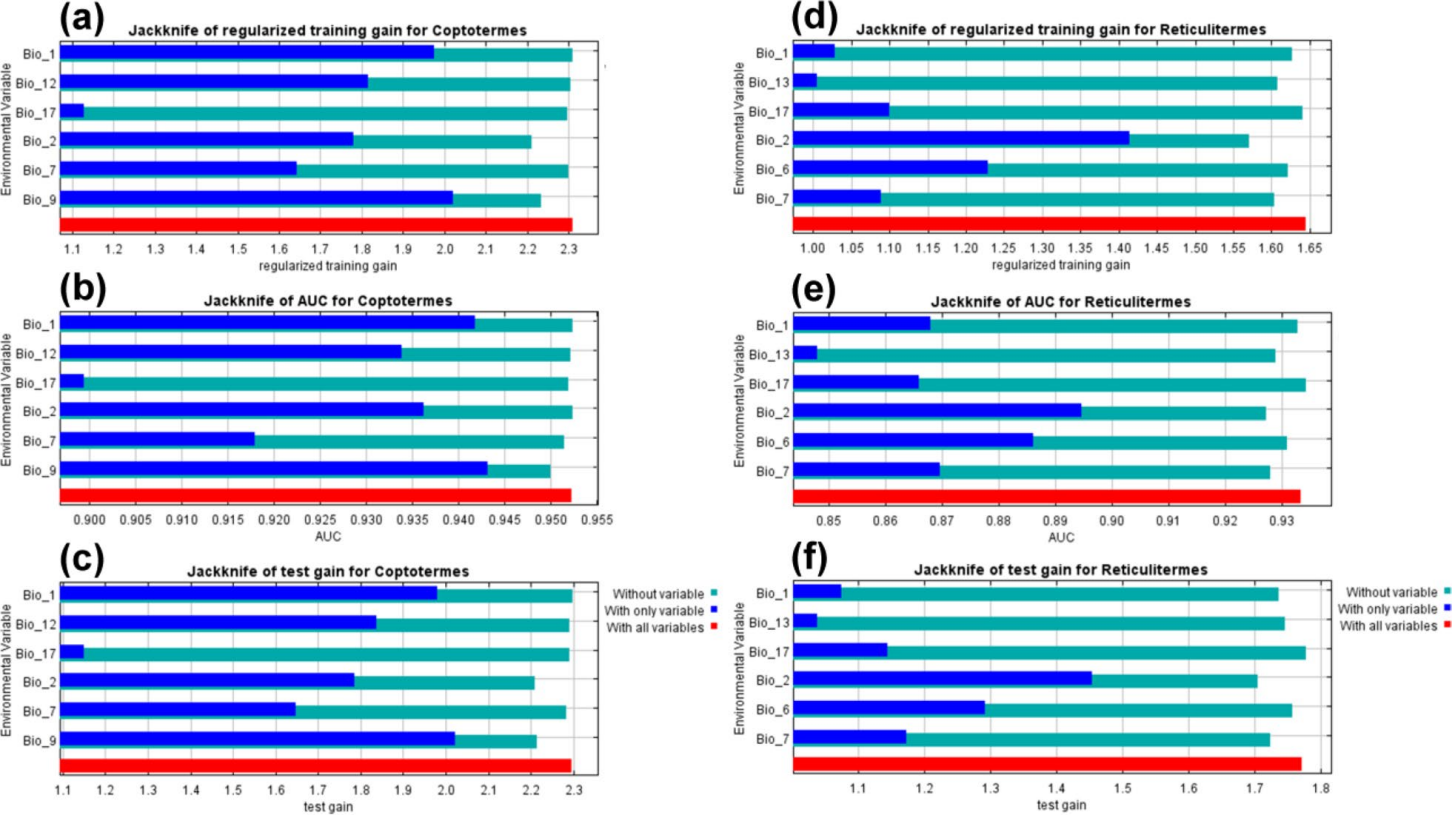
Jackknife test results of Rhinotermitidae (sensu lato) based on the six most influential bioclimatic variables: **a** regularized training gain for *Coptotermes*; **b** AUC values for *Coptotermes*; **c** test gain for *Coptotermes*; **d** regularized training gain for *Reticulitermes*; **e** AUC values for *Reticulitermes*; and **f** test gain for *Reticulitermes*. For the full name of each variable, see Table 1

Figure 5 shows the results of the jackknife test for the six most influential bioclimatic factors utilized in this study for *Coptotermes* and *Reticulitermes*, using the MaxEnt model. The Jackknife analysis for *Coptotermes* indicates that annual mean temperature (Bio1) and mean temperature of driest quarter (Bio9) had the highest gain when employed independently and the greatest decline when excluded (Fig. 5a–c). In the case of *Reticulitermes*, mean diurnal range (Bio2) and minimum temperature of coldest month (Bio6) had the elevated gain when used independently and the highest decrease when excluded (Fig. 5d–f).

The response curves showed a high occurrence chance of *Coptotermes* when Bio1 was above 24 °C, Bio2 was from 4 to 6 °C, temperature annual range (Bio7) was from 8 to 18 °C, Bio9 was from 20 to 28 °C, annual precipitation (Bio12) was greater than 2500 mm, and precipitation of driest quarter (Bio17) was higher than 200 mm (Fig. S6). Furthermore, the response curves illustrated an elevated occurrence probability of *Reticulitermes* when Bio1 was around 22 °C, Bio2 was from 4 to 6 °C, Bio6 was around 12 °C, Bio7 was from 9 to 18 °C, precipitation of wettest month (Bio13) was greater than 400 mm, and Bio17 was higher than 300 mm (Fig. S7). In the final MaxEnt model, the percent contribution of six most influential bioclimatic factors for *Coptotermes* were Bio1 (73.2%), Bio2 (13.6%), Bio12 (4.3%), Bio9 (3.8%), Bio17 (3.6%), and Bio7 (1.5%) (Table S2). Additionally, the percent contribution of the six most important environmental variables for *Reticulitermes* were as follows: Bio17 (31.4%), Bio2 (31%), Bio6 (27.2%), Bio7 (5.3%), Bio13 (3.8%), and Bio1 (1.4%) (Table S3).

### Risk habitats across current and future global warming scenarios

**Fig. 6:**
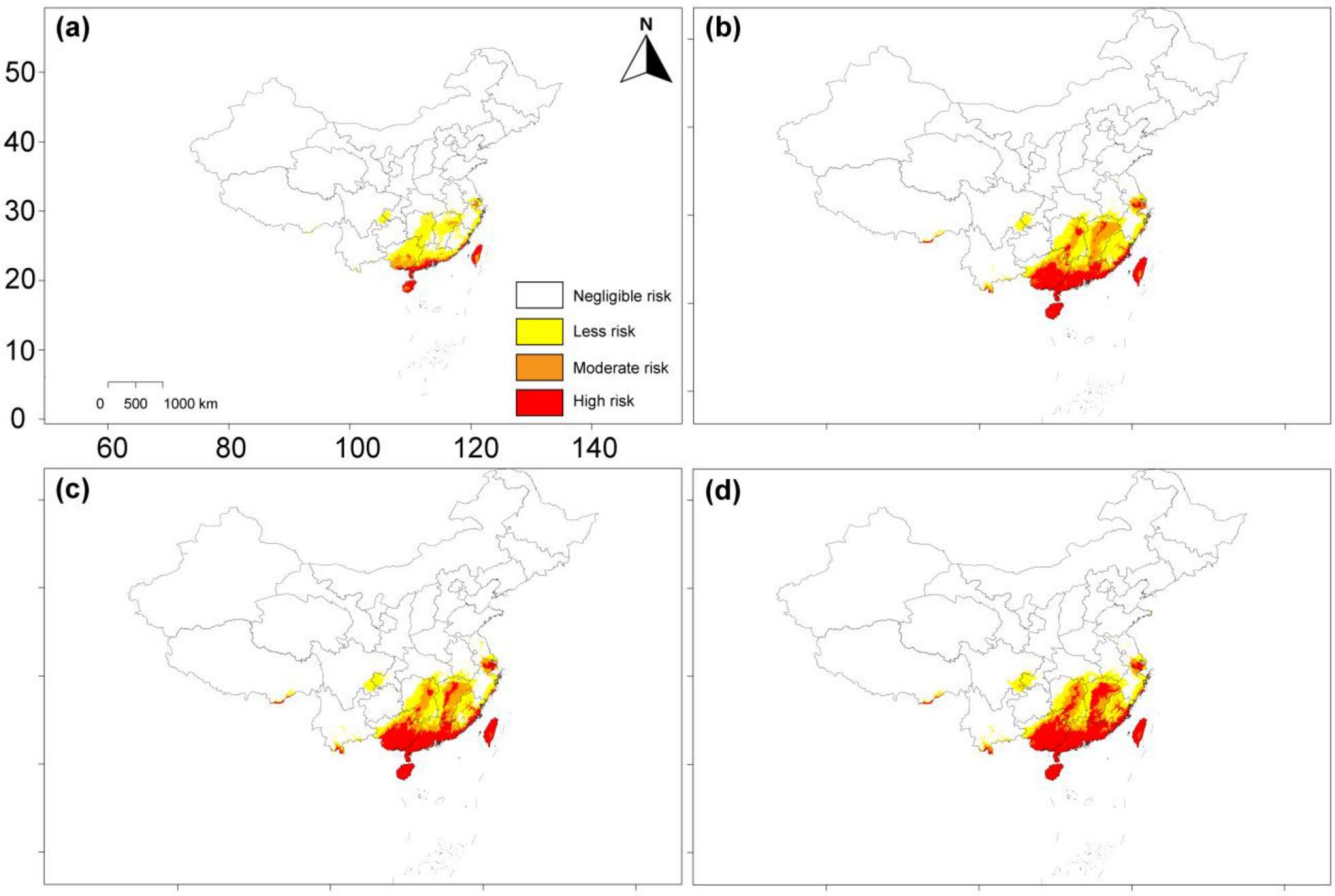
Distribution of *Coptotermes* risk habitats across historical period and future global warming scenarios: **a** Current (1970–2000); **b** SSP2-4.5 (2050s); **c** SSP2-4.5 (2070s); and **d** SSP2-4.5 (2090s)

According to the Current period, the overall risk habitats of *Coptotermes* were mainly found in southern China, particularly the coastal regions (Fig. 6a). High-risk habitats were primarily found in Taiwan Island, Hainan Island, Guangdong Province, Guangxi Province, Macau, and Hong Kong. During the Current period, the overall risk area of *Coptotermes* was 0.73 million km^2^, accounting for around 7.87% of the total study area. Habitats categorized as less-, moderate-, and high-risk regions constitute approximately 67%, 17.8%, and 15% of the total risk habitats, respectively (Fig. 6; Table 2).

**Table 2:** Alterations in Rhinotermitidae (sensu lato; *Coptotermes* and *Reticulitermes*) risk habitats in China under Current (1970–2000) and future global warming scenarios

Habitat suitability	Genus	Current	SSP2-4.5 (2050s)	SSP2-4.5 (2070s)	SSP2-4.5 (2090s)
		Area (million km^2^)	Area (million km^2^)	Area (million km^2^)	Area (million km^2^)
Negligible risk	*Coptotermes*	8.55	8.46	8.45	8.38
Less risk		0.49	0.43	0.40	0.38
Moderate risk		0.13	0.25	0.25	0.24
High risk		0.11	0.36	0.40	0.51
Negligible risk	*Reticulitermes*	7.03	6.80	6.64	6.54
Less risk		1.23	1.37	1.53	1.65
Moderate risk		0.83	1.01	1.01	0.94
High risk		0.19	0.32	0.33	0.37

The risk zones of *Coptotermes* under future global warming scenarios are expected to expand relative to the risk zones across China in the Current period. Figure 6b–d shows *Coptotermes* projected risk zones for the years 2050s, 2070s, and 2090s, based on SSP2-4.5 scenarios. From 1970–2000 to the 2050s, 2070s, and 2090s, the total risk zones are expected to rise to 1.04 million km^2^, 1.05 million km^2^, and 1.13 million km^2^, respectively. Similarly, the high-risk zones are expected to expand continuously from 0.11 million km^2^ in the Current period to 0.36 million km^2^ in the 2050s, 0.40 million km^2^ in the 2070s, and 0.51 million km^2^ in the 2090s, expanding towards new territories in southern China (Table 2).

**Fig. 7:**
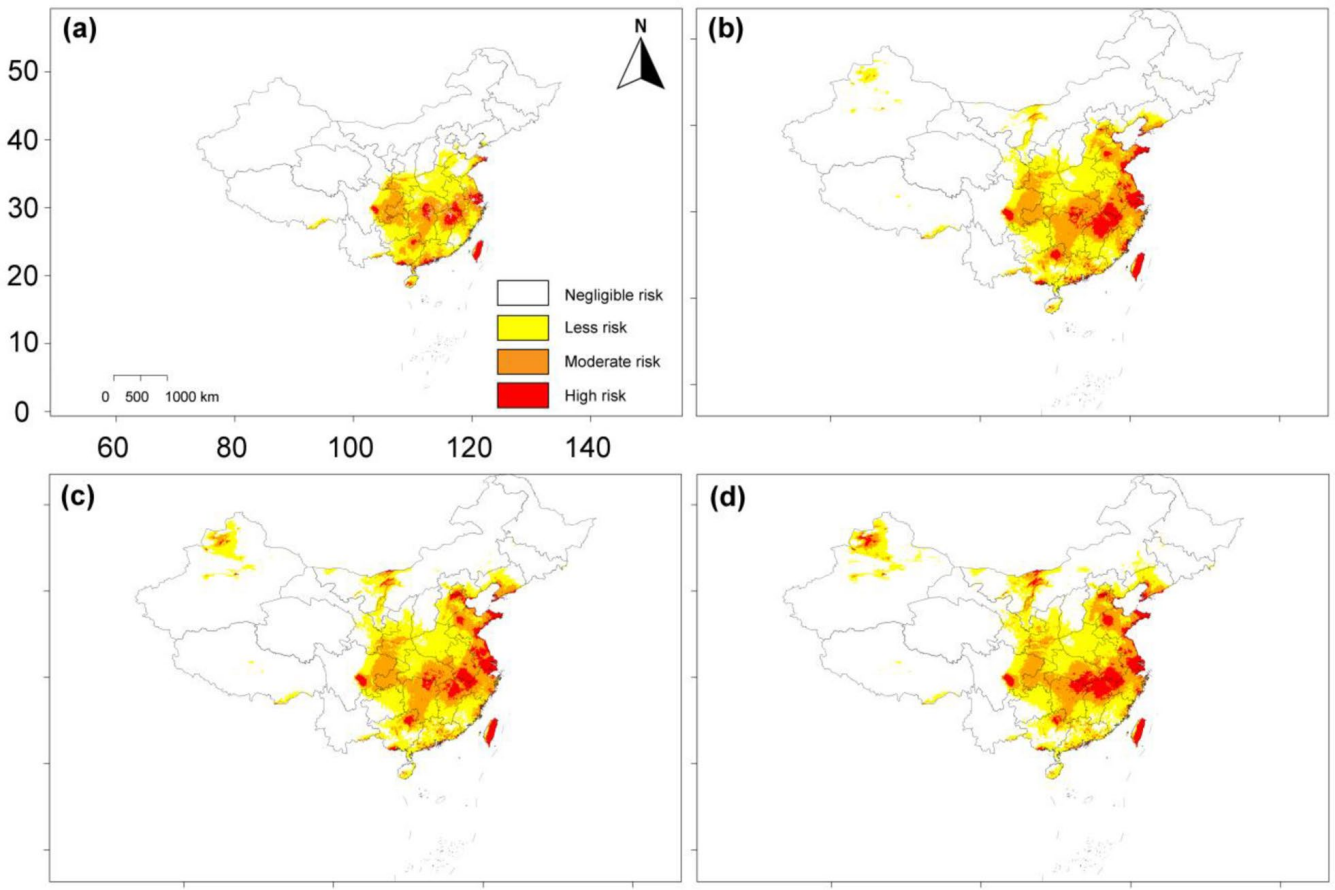
Distribution of *Reticulitermes* risk habitats across historical period and future global warming scenarios: **a** Current (1970–2000); **b** SSP2-4.5 (2050s); **c** SSP2-4.5 (2070s); and **d** SSP2-4.5 (2090s)

Furthermore, as per the Current period, the total risk areas of *Reticulitermes* were mainly found in southern China, Shandong Province, Henan Province, Hebei Province, and Shaanxi Province (Fig. 7a). High-risk habitats were primarily found in Taiwan Island, Hainan Island, Guangdong Province, Macau, Hong Kong, Guangxi Province, Jiangsu Province, Shanghai, Jiangxi Province, Anhui Province, Hunan Province, Hubei Province, Sichuan Province, and coastal regions of Shandong Province. During the Current period, the total risk habitat of *Reticulitermes* was 2.25 million km^2^, accounting for approximately 32% of the overall China. Areas classified as Less-, moderate-, and high-risk regions make up around 54.7%, 36.9%, and 8.4% of the total risk areas, respectively (Fig. 7; Table 2).

The risk areas of *Reticulitermes* throughout future global warming conditions are expected to increase relative to the risk habitats across the study site in the Current period. Figure 7b–d show *Reticulitermes* expected risk areas for the years 2050s, 2070s, and 2090s, based on SSP2-4.5 conditions. From 1970–2000 to the 2050s, 2070s, and 2090s, the overall risk habitats are projected to increase to 2.7 million km^2^, 2.86 million km^2^, and 2.96 million km^2^, respectively. Similarly, the high-risk habitats are projected to increase from 0.19 million km^2^ in the Current period to 0.32 million km^2^ in the 2050s, 0.33 million km^2^ in the 2070s, and 0.37 million km^2^ in the 2090s, expanding towards new regions in southern China, followed by a shift towards northern China and the northwestern part of Xinjiang Province (Table 2).

## Discussion

Termites of the family Rhinotermitidae (sensu lato) [3, 4] pose a constant and complex danger to constructed and natural settings, inflicting ongoing harm to timber structures, forest assets, and cultural heritage buildings [9–11]. The cumulative and recurrent nature of these effects—exacerbated by substantial urban expansion and transport connectivity—imposes significant financial strain on both public infrastructure and private property [10, 11, 26]. National surveys and developmental evaluations have consistently recorded the significant prevalence and extensive spatial distribution of damage caused by termites in China, particularly south of the Yangtze River, with annual direct losses estimated in billions (RMB) [10, 11]. Studies investigated termite species as the primary agents of structural and plant degradation. The covert digging behaviors of termites, which involve interaction with the soil, along with their substantial and resilient colonies, hinder early identification and control efforts [9, 12, 13]. The finding highlights the necessity for geographically detailed risk evaluations and preventive landscape-level mitigation plans guided by the distribution of species and environmental forecasting models.

In the current study, we found that the family Rhinotermitidae (sensu lato) is primarily found in southern China, a distribution closely associated with particular environmental factors characterizing the study area's humid and warm environment [3, 4, 8, 46]. This study identified temperature (*Coptotermes*: Bio1, Bio2, Bio7, Bio9; *Reticulitermes*: Bio1, Bio2, Bio6, Bio7) and precipitation (*Coptotermes*: Bio12, Bio17; *Reticulitermes*: Bio13, Bio17) as the principal determinants influencing the habitat suitability and distribution of Rhinotermitidae (sensu lato) in China. These factors together create climate conditions that are suitable for *Coptotermes* and *Reticulitermes* to live, gather, and grow their colonies. For example, mild temperature changes (Bio1, Bio2, Bio6, Bio7, and Bio9) keep biological activity going all year, and high yearly and seasonal rainfall (Bio12, Bio13, and Bio17) keeps the soil moist enough for termites to nest underground and break down wood. Furthermore, *Coptotermes* achieves optimal stability in warmer environments (Bio1 > 24 °C) and lower precipitation thresholds (Bio17 > 200 mm), reflecting its enhanced resistance to annual mean temperature and seasonal aridity. Conversely, *Reticulitermes* demonstrates optimal adaptability at somewhat lower annual mean temperatures (Bio1 ~ 22 °C) and elevated seasonal precipitation levels (Bio17 > 300 mm), indicating its significant reliance on humidity and tolerance to cold temperatures [47–50]. Our results align with studies that consistently identify temperature- and moisture-associated factors as primary determinants of termite distributions, expansion, and increased invasive danger [17, 21]. Termite species, including *C. formosanus* and *R. flavipes*, exhibited a preference for low to moderate temperatures and elevated humidity, with death rates significantly increasing under extreme heat and arid conditions [21]. Additionally, research demonstrates that temperature and humidity influence the distribution and expansion of *C. formosanus*, *C. gestroi*, *Coptotermes havilandi*, *R. flavipes*, and *Reticulitermes grassei* [17], hence validating the significance of bioclimatic variables in constraining the northern habitat limits of *Coptotermes* and *Reticulitermes* in China. Collectively, these findings indicate that the mild, relatively stable, and moist environment of southern China creates ideal circumstances for the ongoing existence and proliferation of both genera, which reinforces the importance of these ecological limits in informing future surveillance and targeted termite control approaches.

China has undergone climate change over the past two decades [51]. The National Center for Atmospheric Research indicates that temperatures in China may increase by 2.8–5.3 °C during the twenty-first century [52]. In this study, based on Rhinotermitidae (sensu lato) occurrence data, the MaxEnt model indicates a substantial increase in the risk habitats of *Coptotermes* and *Reticulitermes* throughout China, demonstrating both an increase in existing areas (southern China) and a northern migration into previously (1970–2000) negligible- and less-risk regions. The regions classified as less-, moderate-, and high-risk for *Coptotermes* are projected to expand from 0.73 million km^2^ in the Current period to 1.13 million km^2^ in the future (SSP2-4.5; 2090s). Consequently, *Coptotermes* risk habitats are expected to increase further in the southern region of the study area, particularly in Hunan Province, Jiangxi Province, Fujian Province, and Shanghai. In the case of *Reticulitermes*, the areas categorized as less-, moderate-, and high-risk are expected to increase from 2.25 million km^2^ in the Current period to 2.96 million km^2^ in the future (SSP2-4.5; 2090s). Therefore, *Reticulitermes* risk regions are projected to increase further in southern China, followed by a shift towards northern China and the northwestern part of Xinjiang Province.

Although environmental appropriateness is essential for growth, the effective invasion of these places relies on dispersion processes that slightly differ between the two genera due to their different nesting biology [17, 53–56]. *Coptotermes* species make moisture-retaining carton nests that enable colonies to thrive freely within infested wood, thereby augmenting their potential for anthropogenic dispersal across extensive distances through infected timber, wood-derived products, and various transportation methods, ultimately facilitating the establishment of ecologically favorable habitats [17, 19, 53, 55–58]. Conversely, *Reticulitermes* species rely on soil that requires constant moisture and often disseminate over small distances by alate swarming, hence restricting extensive colonization to areas adjacent to established populations [20, 54–57, 59]. These biological variations indicate that the establishment of newly appropriate areas, especially in northern China, is predominantly influenced by human-facilitated distribution in *Coptotermes*, whereas the spread in *Reticulitermes* is anticipated to depend mainly on spontaneous swarming events.

Furthermore, studies have predicted that the distribution range of termites will expand due to global warming [17, 26]. Multiple processes support this pattern. Increasing temperatures alleviate cold-related limitations, which may extend active durations for colony development, migration, and longevity [12, 17, 18, 21, 26]. Higher temperatures and a longer warm season may speed up biological and metabolic processes, which leads to faster development and higher reproductive output under optimal threshold scenarios [12, 17, 26, 60]. For example, termite queens have been shown to lay more eggs when their nests have low-O₂ and high-CO₂ levels [61]. Concurrently, moisture-retaining precipitation patterns alleviate drought-related colony tension, hence promoting larval development and worker selection [17, 26, 62]. The increased availability of humidity and elevated temperatures facilitates faster attainment of sexual maturity in colonies, leading to the establishment of larger secondary nests, hence enhancing the number of colonies and broadening spatial distribution [17, 26, 63]. Research demonstrates that termite intake and colony activities significantly escalate with temperature—wood-decay speeds surpass 6.8 times for every 10 °C increase—suggesting an expanded resource base for development and reproduction in hotter climates [12]. These mechanisms together connect the environmental improvement of biological, developmental, and environmental limitations to the anticipated increase and shift of *Coptotermes* and *Reticulitermes* risk zones in China, offering mechanistic validation for our geographical forecasts and indicating a pressing need for proactive surveillance in the newly emerging risk areas.

Considering the anticipated northward extension and strengthening of *Coptotermes* and *Reticulitermes* areas in China due to global warming, it is essential for government organizations, insect prevention officials, and infrastructure owners to implement a comprehensive and proactive prevention strategy. Regional treatment programs that utilize chitin-synthesis inhibitors (CSI) or growth-control baits across numerous colonies have proven highly effective in reducing the termite threat by eradicating a significant number of active colonies within treated areas, rather than just addressing isolated outbreaks [64]. Soil barrier applications employing non-repellent insecticides, like chlorantraniliprole, must be emphasized in highly susceptible extension areas revealed by our modeling that might shortly attain environmental suitability for Rhinotermitidae (sensu lato) colonization [3, 4, 65]. Proactive surveillance systems, such as sensor networks, sound detectors, and smart traps, incorporated into urban and rural border frameworks, facilitate the swift identification of emerging outbreaks prior to their escalation [66, 67], a technique particularly vital in increasingly appropriate northern regions. Our climate fit maps should inform risk-specific monitoring and prioritization: local authorities ought to commit funds to towns projected to experience significant changes in the compatibility of bioclimatic variables despite currently minimal termite occurrence. People's education and building laws must complement chemical and inspection measures—contractors in particularly susceptible regions should be mandated to install physical obstacles (steel mesh, concrete grouting) and termite-resistant products during the building process, therefore diminishing potential susceptibility [68]. Ultimately, adaptive planning should be institutionalized: as our model indicates, changing appropriate regions, laws, and procedure implementation must be revised periodically (e.g., every 5–10 years), integrating new environmental and surveillance records and facilitating flexible resource reallocation instead of fixed, southern-exclusive programs. In summary, integrating geographically informed monitoring, planned physical and chemical measures, and legislative and social involvement provides a thorough approach to managing the imminent proliferation of Rhinotermitidae (sensu lato) in China.

In this study, we used 19 environmental factors to estimate the occurrence and prospective spread of *Coptotermes* and *Reticulitermes* across China; nevertheless, it is constrained by many important limitations that should be covered up in future studies. Our investigation excluded factors like biotic relationships (competition, predation, and host-tree accessibility) and human-caused variables (usage of land, construction resources, and urban heat islands). However, prior research warns that similar species distribution models could underestimate appropriateness in contexts where these non-environmental variables prevail [31, 32, 41]. We also did not include edaphic (soil type, pH, and texture), physical habitat (timber mass, forest cover, and human habitation), and dispersal blocking (transport networks and human-induced introductions) factors, every one of which is acknowledged as significant in termite and other organism distribution modeling [66, 67, 69, 70]. Despite adhering to norms in our MaxEnt validation, modeling over extensive areas continues to pose the danger of sampling error [71]. Ultimately, although our findings indicate a rise in environmental appropriateness, it is impossible to independently forecast actual harm occurrence, colony formation success, or management outcomes; thus, subsequent fieldwork, continuous surveillance of termite colony trends, and the incorporation of economic and social indicators are imperative. Additionally, analyses at a finer (species) taxonomic level are necessary to obtain truly actionable risk maps. Further studies must incorporate detailed non-environmental variables as mentioned earlier, specifically simulate spread and interaction limitations, examine habitat variations through experimental physiology and genomics, and align species distribution model results with actual invasion and harm evaluations to facilitate specific control measures.

## Conclusion

In this study, we assessed the geographic distribution trend and hotspots of the family Rhinotermitidae (sensu lato) throughout China. The distribution and species richness hotspots of both *Coptotermes* and *Reticulitermes* are mainly located in southern China. The MaxEnt model was used to predict possible risk ranges of *Coptotermes* and *Reticulitermes* in the Current period and under future climate change conditions, utilizing six major bioclimatic variables for each genus. The variable Bio1 predominantly affects the distribution of *Coptotermes* in China, while the variables Bio2 and Bio17 mainly affect the distribution of *Reticulitermes*. Compared to the Current period, the prediction outcomes for climate change conditions SSP2-4.5 indicate that the overall risk zones for *Coptotermes* and *Reticulitermes* are anticipated to rise in the 2050s, 2070s, and 2090s, with a spread towards northern China. These results indicate an increase in the diversity of Rhinotermitidae (sensu lato) and the potential for future colonization of novel areas. Our results establish a vital foundation for effective, geographically oriented termite control measures in China, allowing policymakers to predict and alleviate the northward proliferation of Rhinotermitidae (sensu lato) due to global warming in the future.

Besides the climatic variables used in this study, other factors that may affect the distribution of Rhinotermitidae (sensu lato) include forest cover, soil properties, physical habitat, dispersal barriers, and anthropogenic activities. In future research, these factors can be analyzed to enhance the accuracy of Rhinotermitidae (sensu lato) distribution predictions.

## Supplementary Information

Additional file1 (DOCX 1502 kb)

## Data Availability

The dataset supporting the conclusions of this article are included within the article and its additional files.
