# Supplementary material for: Assessing the invasive risk of Rhinotermitidae in China under current and future global warming scenarios using the MaxEnt model
Source: Front Zool. 2026 Feb 21;23:10. doi: 10.1186/s12983-026-00600-x (PMC13032235; doi:10.1186/s12983-026-00600-x)
Supplement: Supplementary file 1 — Additional file1 (DOCX 1502 kb) [file 12983_2026_600_MOESM1_ESM.docx]

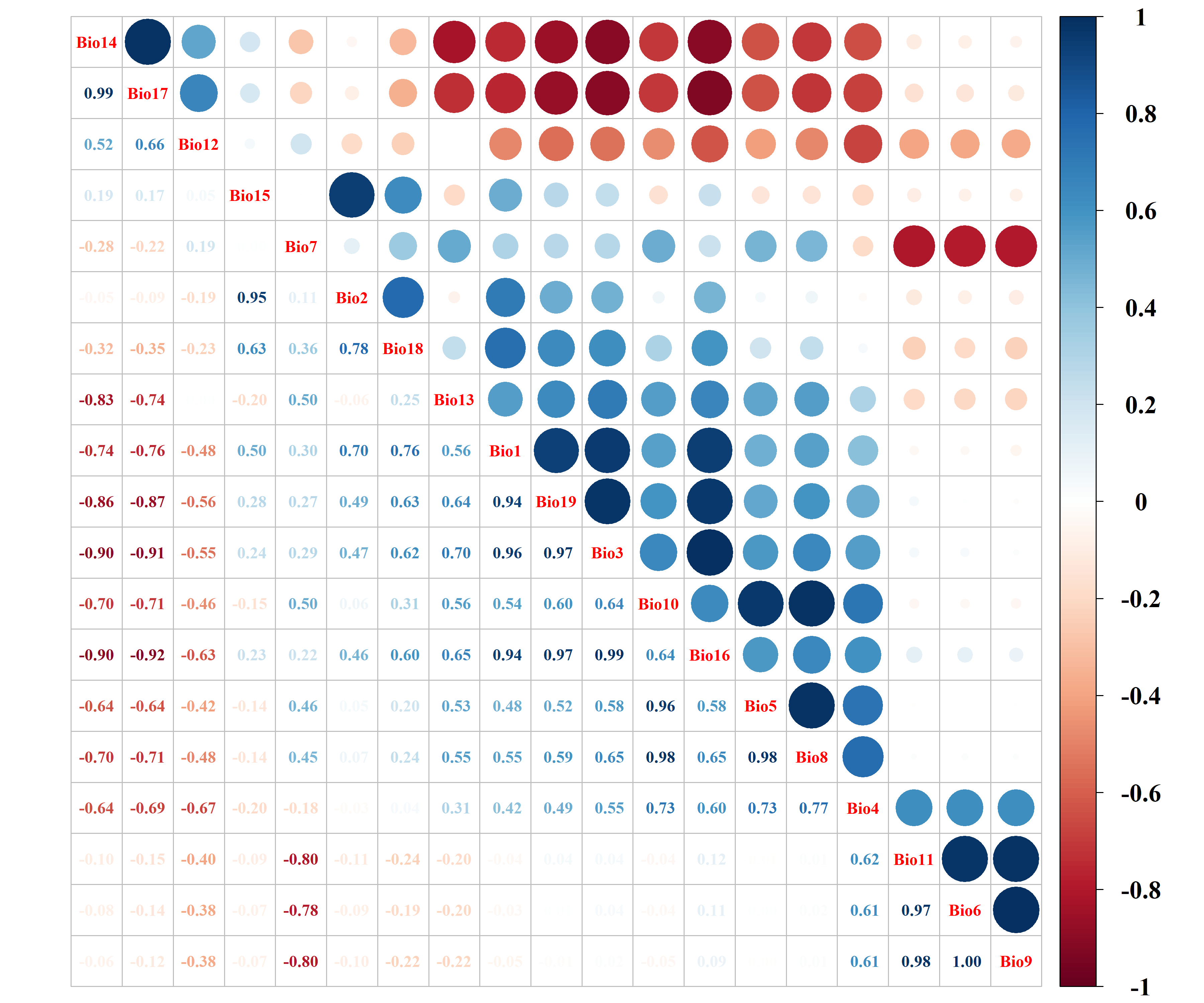


**Fig. S1** Correlation analysis of bioclimatic variables with *Coptotermes* across China. Note that the lower left portion represents the correlation value *r*, varying from −1 to 1. The positive numbers show positive associations, negative numbers show negative associations, and 0 shows no correlation. The higher the value of |*r*|, the more significant the association. The upper right portion is a graphical transformation of correlation values. Positive associations are displayed in blue and negative associations in red color. Color intensity and size of the circle are proportional to the correlation coefficients (refer to the legend on the right).


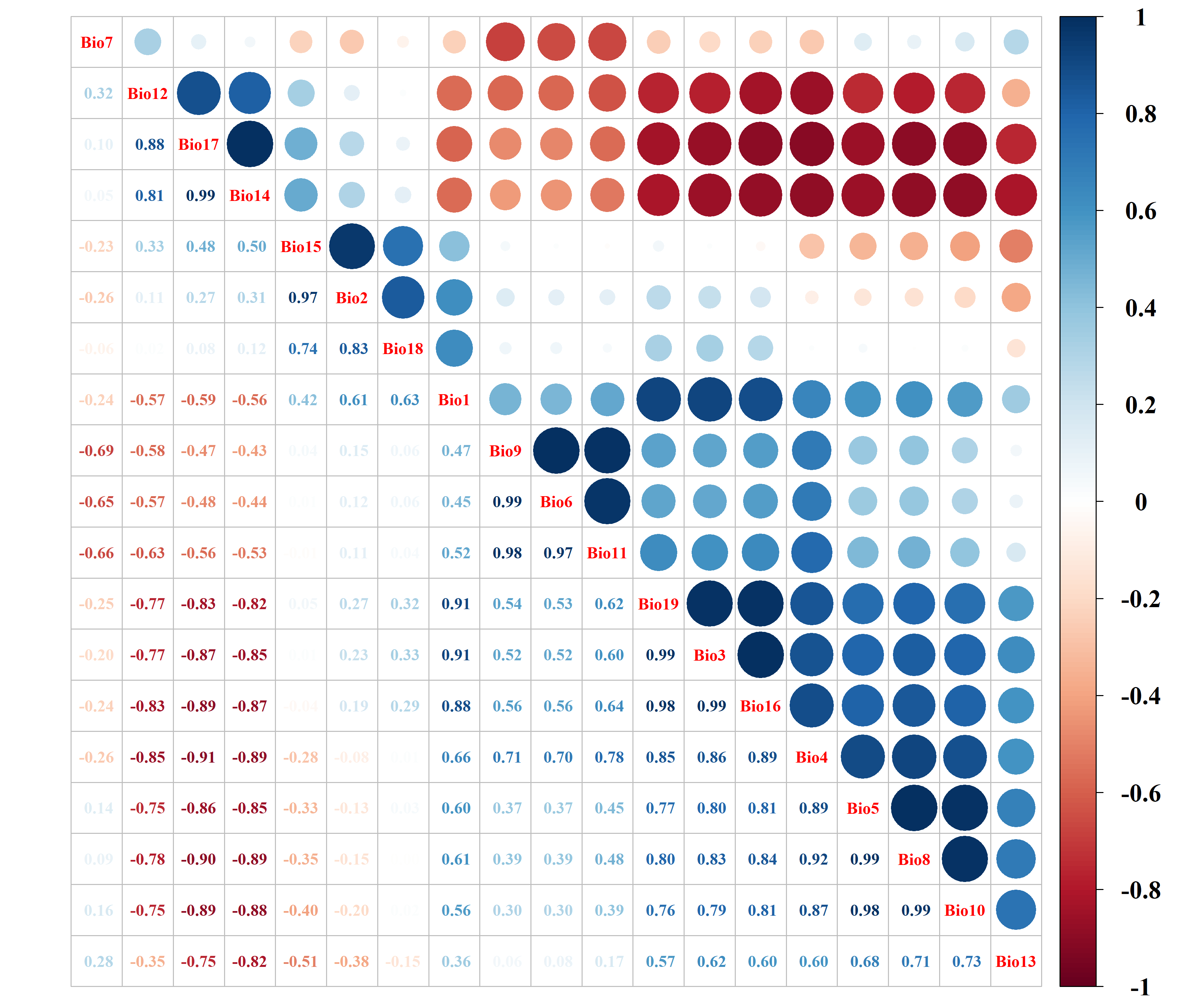


**Fig. S2** Correlation analysis of bioclimatic variables with *Reticulitermes* across China. Note that the lower left portion represents the correlation value *r*, varying from −1 to 1. The positive numbers show positive associations, negative numbers show negative associations, and 0 shows no correlation. The higher the value of |*r*|, the more significant the association. The upper right portion is a graphical transformation of correlation values. Positive associations are displayed in blue and negative associations in red color. Color intensity and size of the circle are proportional to the correlation coefficients (refer to the legend on the right).


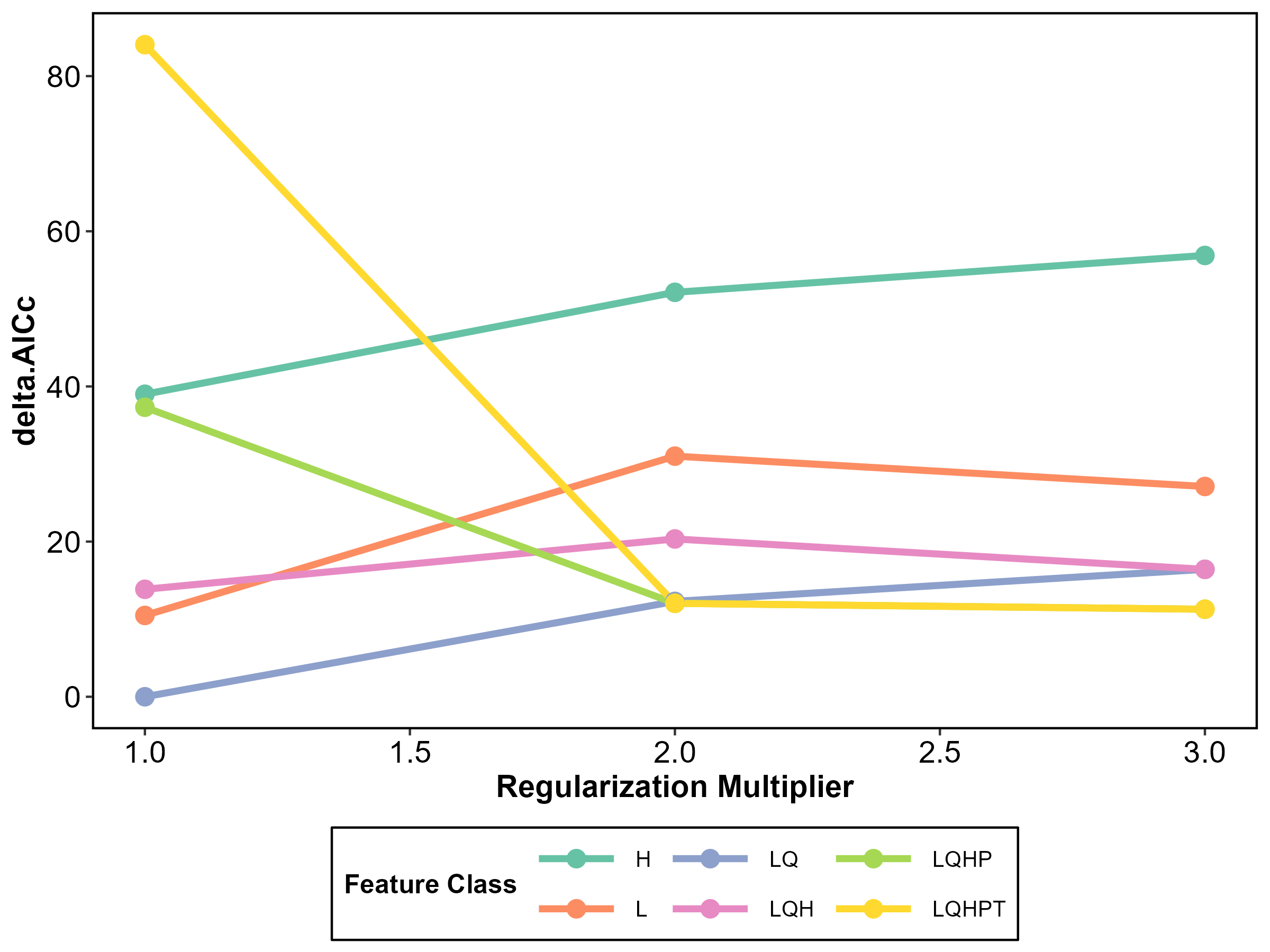


**Fig. S3** The delta AICc-value of the model under user-specified range of regularization multiplier (RM) and feature combinations (FCs) for *Coptotermes*.


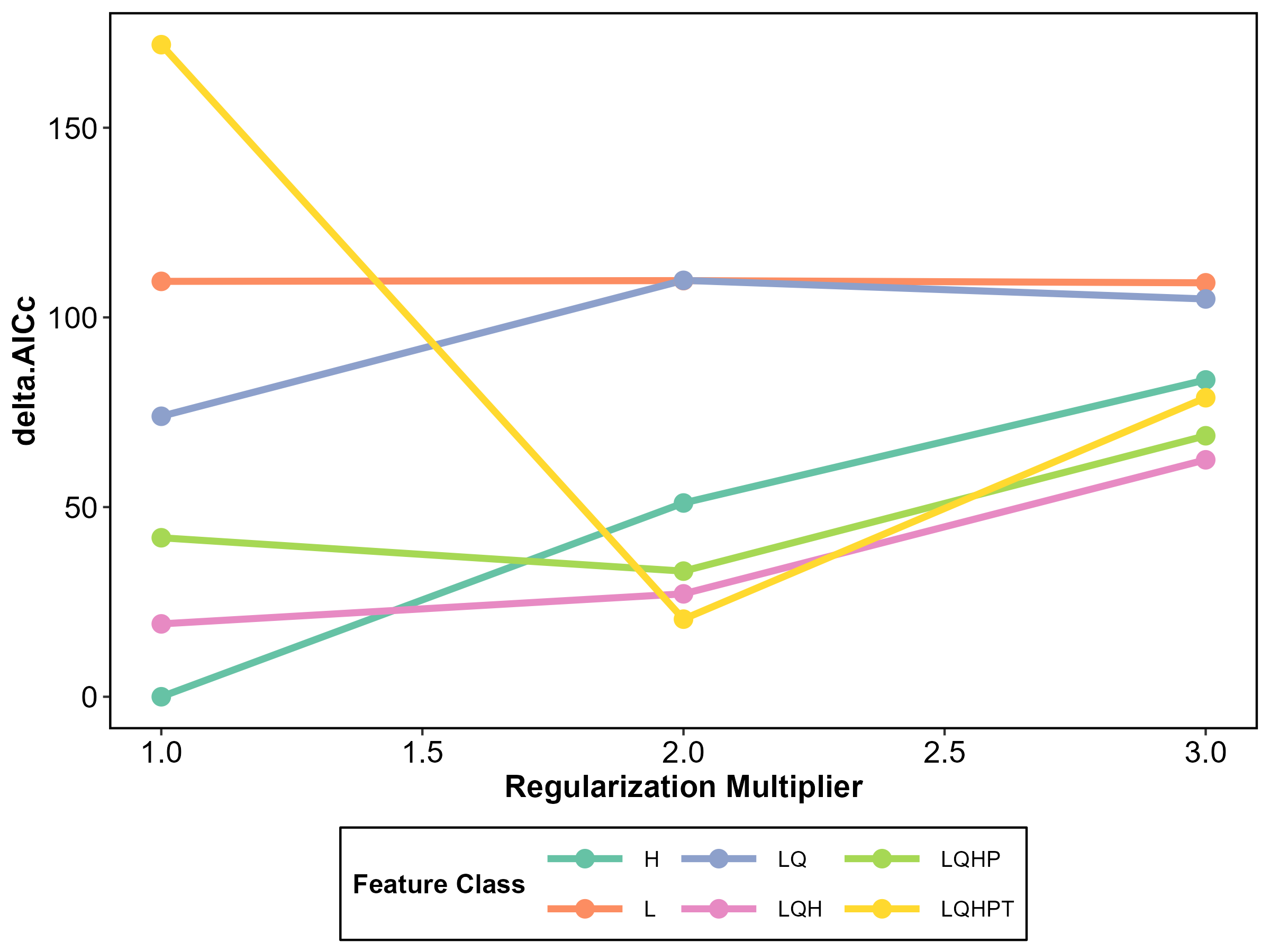


**Fig. S4** The delta AICc-value of the model under user-specified range of regularization multiplier (RM) and feature combinations (FCs) for *Reticulitermes*.


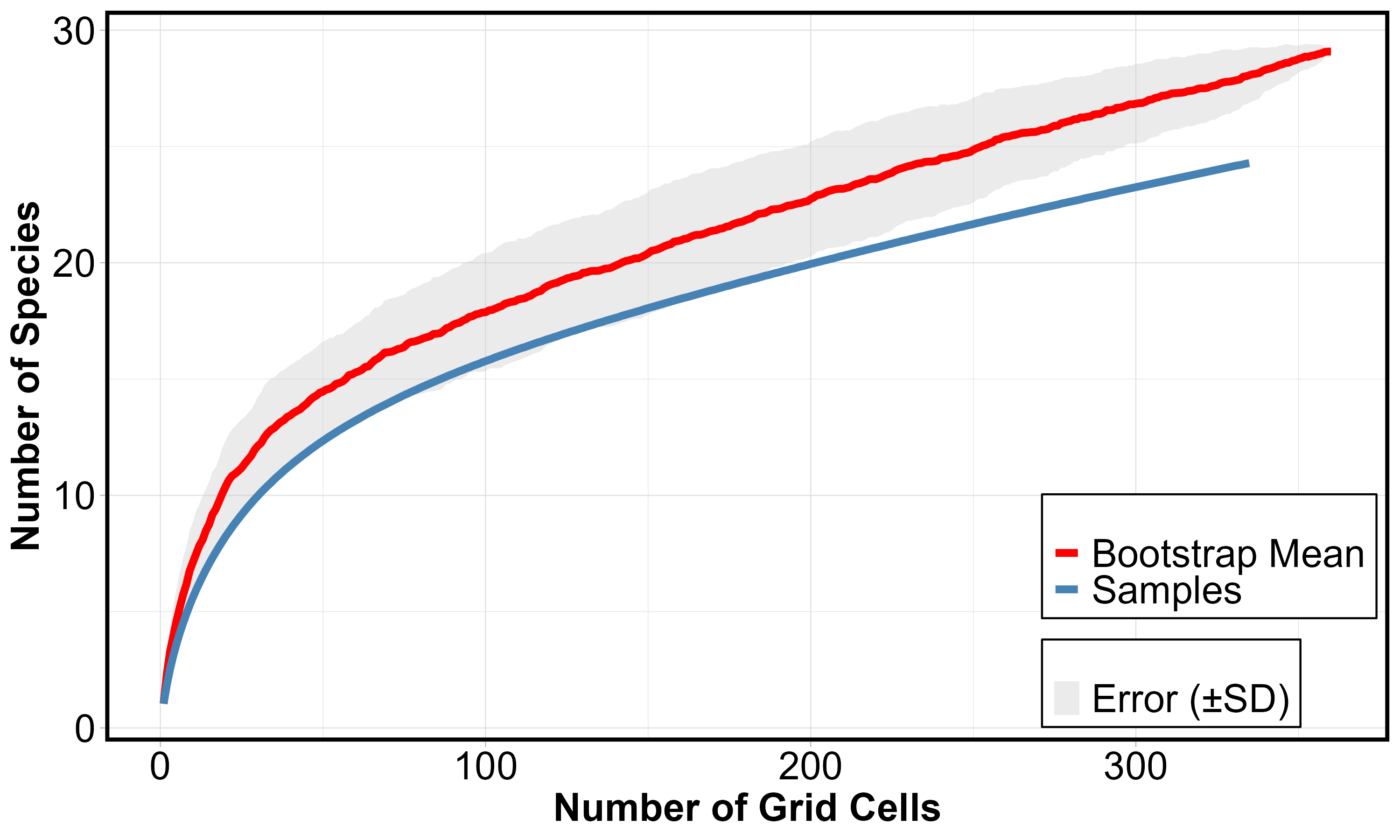


**Fig. S5** Species accumulation curves for Rhinotermitidae based on bootstrap mean values.


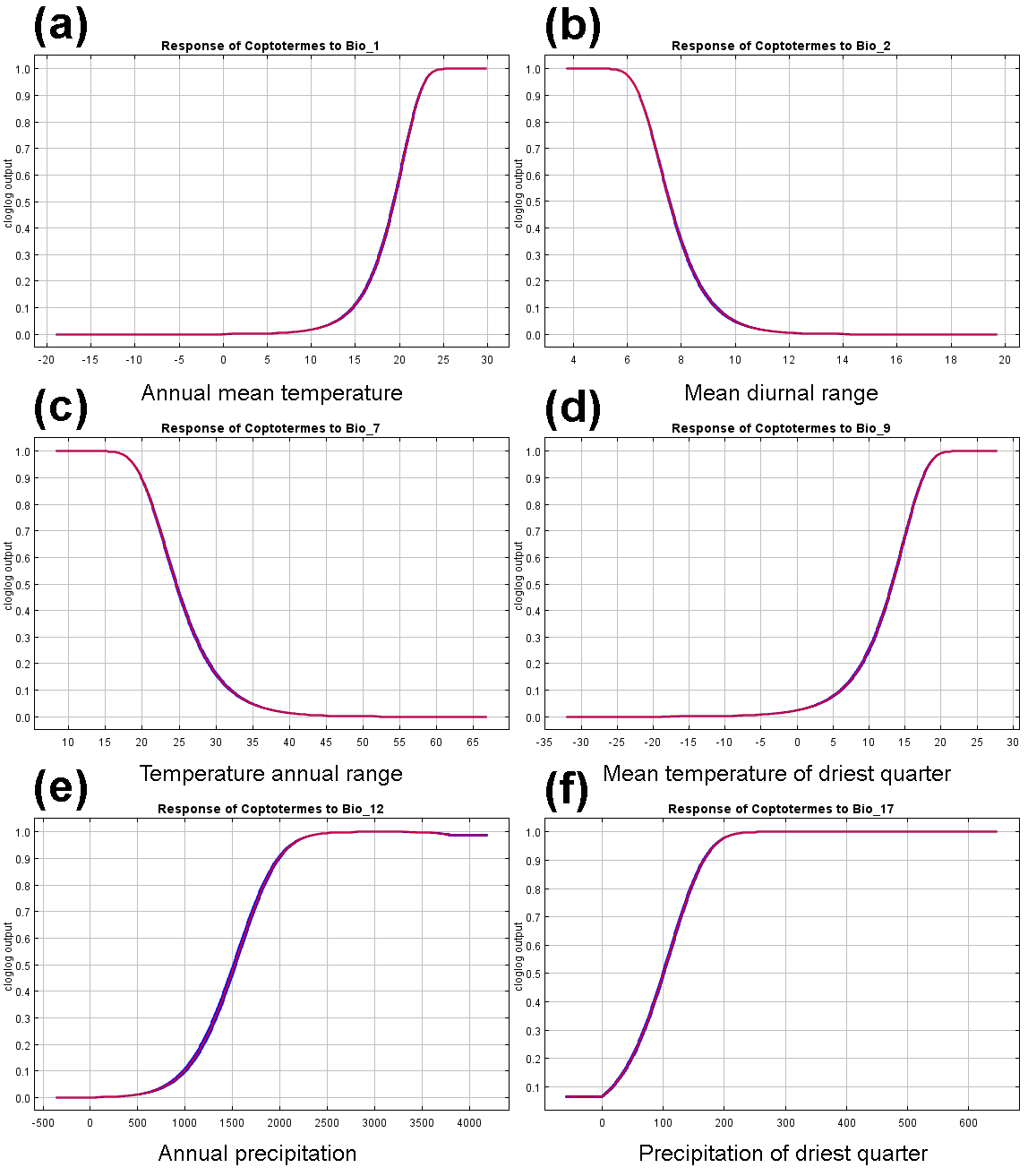


**Fig. S6** Response curves of the six most influential bioclimatic factors influencing *Coptotermes* distribution.


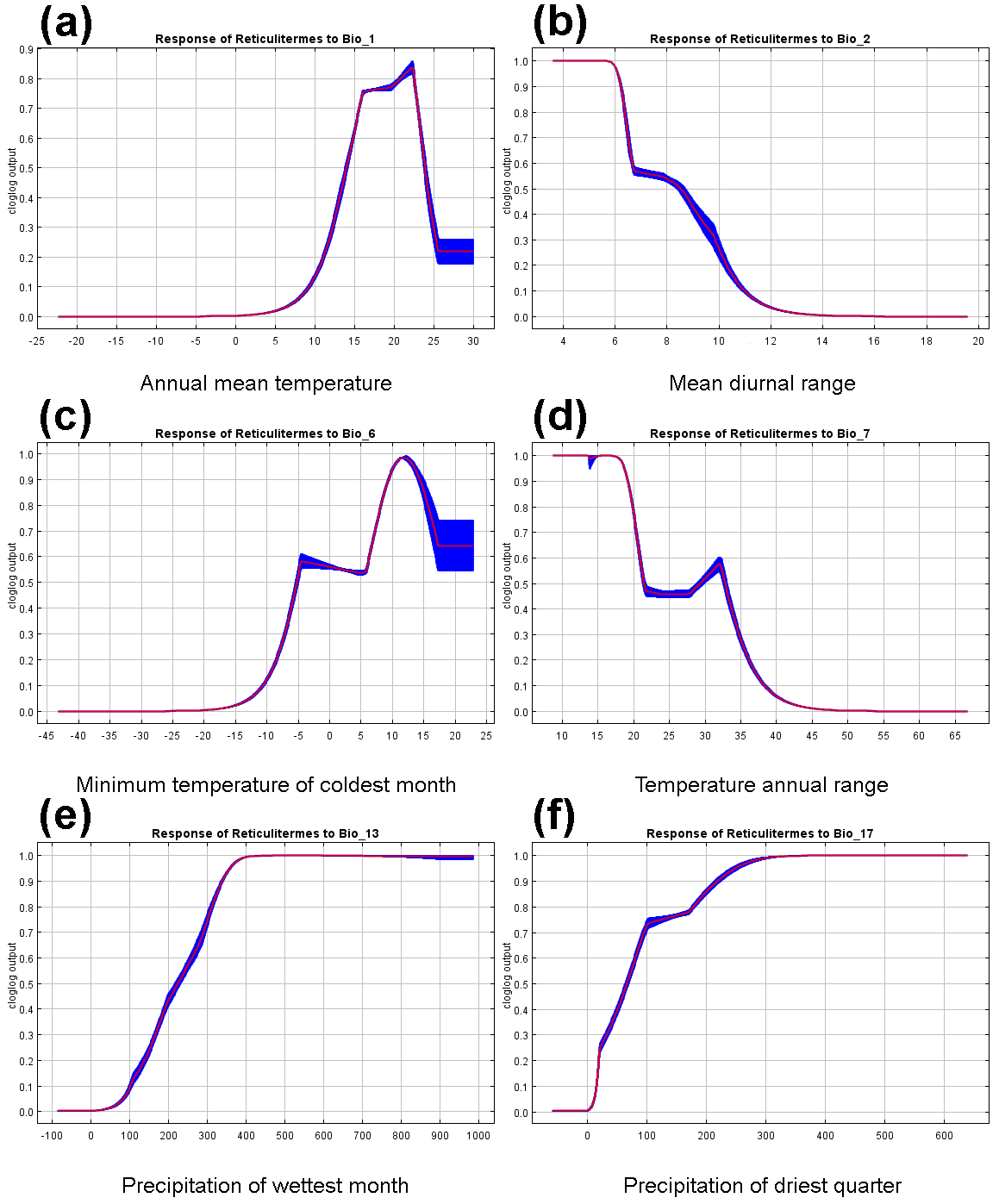


**Fig. S7** Response curves of the six most influential bioclimatic factors influencing *Reticulitermes* distribution.

**Table S1.** Rhinotermitidae species list used in the current study.

| **Species List** |
| --- |
| *Coptotermes curvignathus* |
| *Coptotermes elisae* |
| *Coptotermes formosanus* |
| *Coptotermes gestroi* |
| *Coptotermes suzhouensis* |
| *Coptotermes testaceus* |
| *Reticulitermes aculabialis* |
| *Reticulitermes affinis* |
| *Reticulitermes chinensis* |
| *Reticulitermes curvatus* |
| *Reticulitermes dichrous* |
| *Reticulitermes flaviceps* |
| *Reticulitermes flavipes* |
| *Reticulitermes grandis* |
| *Reticulitermes guangzhouensis* |
| *Reticulitermes kanmonensis* |
| *Reticulitermes khaoyaiensis* |
| *Reticulitermes labralis* |
| *Reticulitermes leptomandibularis* |
| *Reticulitermes ovatilabrum* |
| *Reticulitermes periflaviceps* |
| *Reticulitermes speratus* |
| *Reticulitermes tricholabralis* |
| *Reticulitermes chinensis* |

**Table S2.** Percent contribution of top six bioclimatic variables influencing *Coptotermes* distribution in China using MaxEnt model.

| **Variable** | **Percent contribution** |
| --- | --- |
| Bio1 | 73.2 |
| Bio2 | 13.6 |
| Bio12 | 4.3 |
| Bio9 | 3.8 |
| Bio17 | 3.6 |
| Bio7 | 1.5 |

**Table S3.** Percent contribution of top six bioclimatic variables influencing *Reticulitermes* distribution in China using MaxEnt model.

| **Variable** | **Percent contribution** |
| --- | --- |
| Bio17 | 31.4 |
| Bio2 | 31 |
| Bio6 | 27.2 |
| Bio7 | 5.3 |
| Bio13 | 3.8 |
| Bio1 | 1.4 |
